# Monkeypox: epidemiology, pathogenesis, treatment and prevention

**DOI:** 10.1038/s41392-022-01215-4

**Published:** 2022-11-02

**Authors:** Yong Huang, Li Mu, Wei Wang

**Affiliations:** Department of Biotherapy, State Key Laboratory of Biotherapy and Cancer Center, West China Hospital, Sichuan University, Chengdu, China

**Keywords:** Microbiology, Infectious diseases

## Abstract

Monkeypox is a zoonotic disease that was once endemic in west and central Africa caused by monkeypox virus. However, cases recently have been confirmed in many nonendemic countries outside of Africa. WHO declared the ongoing monkeypox outbreak to be a public health emergency of international concern on July 23, 2022, in the context of the COVID-19 pandemic. The rapidly increasing number of confirmed cases could pose a threat to the international community. Here, we review the epidemiology of monkeypox, monkeypox virus reservoirs, novel transmission patterns, mutations and mechanisms of viral infection, clinical characteristics, laboratory diagnosis and treatment measures. In addition, strategies for the prevention, such as vaccination of smallpox vaccine, is also included. Current epidemiological data indicate that high frequency of human-to-human transmission could lead to further outbreaks, especially among men who have sex with men. The development of antiviral drugs and vaccines against monkeypox virus is urgently needed, despite some therapeutic effects of currently used drugs in the clinic. We provide useful information to improve the understanding of monkeypox virus and give guidance for the government and relative agency to prevent and control the further spread of monkeypox virus.

## Introduction

**Fig. 1:**
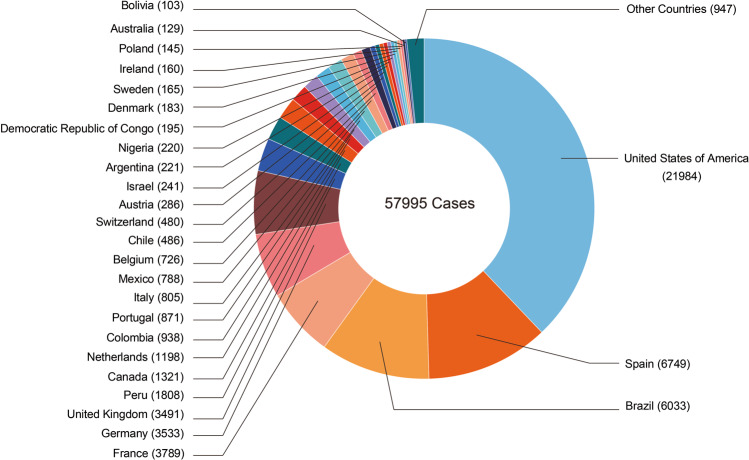
Geographical distribution of confirmed monkeypox cases during the outbreak between January and September 2022. Confirmed cases include those laboratory-confirmed as monkeypox virus and may include cases only confirmed as orthopoxvirus. Data presented as of 12 September 2022 were obtained from CDC. Diagram generated with GraphPad Prism 9

Monkeypox, an endemic disease in both West and Central Africa, before 2022 outbreak, a few cases were reported outside of Africa and these were associated with imports from endemic countries.^1–5^ However, following the initial case diagnosed in the United Kingdom, the number of monkeypox infection have dramatically increased.^3,4,6–8^ We are now facing the first multiple countries outbreak most with no clear epidemiological links to the endemic countries.^9,10^ World Health Organization (WHO) Director-General has announced the ongoing monkeypox outbreak a Public Health Emergency of International Concern (PHEIC).^11,12^ The US Centers for Disease Control and Prevention (CDC) reported that as of 13 September 2022 more than 57,995 confirmed cases had been documented in 100 countries/territories^13^ (Fig. 1 and Table 1).

**Table 1:** 2022 monkeypox global outbreak

Country	Cases^a^	Country	Cases	Country	Cases	Country	Cases
United States of America	21984	Poland	145	Estonia	10	Qatar	3
Spain	6749	Australia	129	India	10	Congo	3
Brazil	6033	Bolivia	103	Central African Republic	8	Venezuela	3
France	3789	Norway	82	Lebanon	8	The Bahamas	2
Germany	3533	Ghana	76	Saudi Arabia	8	Cuba	2
United Kingdom	3491	Hungary	71	Cameroon	7	Georgia	2
Peru	1808	Greece	66	Thailand	7	Greenland (Den.)	2
Canada	1321	Ecuador	59	Bulgaria	5	Guyana	2
Netherlands	1198	Czechia	58	Cyprus	5	Liberia	2
Colombia	938	Luxembourg	53	Lithuania	5	Moldova	2
Portugal	871	Slovenia	45	New Zealand	5	Montenegro	2
Italy	805	Romania	36	South Africa	5	South Korea	2
Mexico	788	Malta	33	Uruguay	5	South Sudan	2
Belgium	726	Serbia	31	Andorra	4	Sudan	2
Chile	486	Finland	30	Honduras	4	Barbados	1
Switzerland	480	Croatia	27	Japan	4	Egypt	1
Austria	286	Dominican Republic	21	Latvia	4	El Salvador	1
Israel	241	Singapore	16	Philippines	4	Indonesia	1
Argentina	221	United Arab Emirates	16	China	4	Iran	1
Nigeria	220	Slovakia	14	Benin	3	Paraguay	1
Democratic Republic of Congo	195	Guatemala	12	Bosnia and Herzegovina	3	Russia	1
Denmark	183	Iceland	12	Costa Rica	3	Turkey	1
Sweden	165	Panama	12	Monaco	3		
Ireland	160	Jamaica	11	Morocco	3		

^a^Data updated on September 13, 2022 (Source: CDC)

Monkeypox virus belongs to the Poxviridae family’s Orthopoxvirus (OPV) genus, along with variola virus (VARV, also known as smallpox), vaccinia virus (VACV), camelpox virus (CMPV), and cowpox virus (CPXV),^14–16^ all of which are pathogenic for humans. There was 96.3% identity between the monkeypox virus genome’s central region, which encodes essential enzymes and structural proteins, and VARVs, which means they are highly genetically similar.^17,18^ In 1980, the WHO certified that smallpox had been eradicated worldwide after successful vaccination.^19–22^

Monkeypox virus has double-stranded DNA (dsDNA) as its genetic material that resembles brick-like particles ranging in size from 200–250 nm in diameter and thus can be seen by electron microscopy (magnified approximately ×10,000).^23,24^ In monkeypox virus, there are two genetic clades: West Africa (WA, Clade II) clade and Congo Basin (CB, Clade I) clade,^25,26^ the latter is also known as the Central African (CA) clade and more severe after infection.^27–32^ In 1958, monkeypox virus was first discovered in Copenhagen among laboratory monkeys, and its first monkeypox virus infection of a human occurred in 1970 in the Democratic Republic of Congo (DRC) in a 9-month-old child, with suspected smallpox.^33–35^ Monkeypox is endemic in both West Africa and Central Africa since 1970, with a mortality rate between 1 and 10%.^36,37^ Sequencing analysis of the current outbreak of monkeypox indicates that the virus is Western African in origin.^38–43^

Recently, the number of patients of monkeypox has increased dramatically, provoking great concern.^44–49^ For this epidemic to be controlled, monkeypox virus must be better understood and re-evaluated.^50,51^ Here, we review the recent investigations on monkeypox, including epidemiology of monkeypox, monkeypox virus reservoirs, novel transmission patterns, mutations and mechanisms of viral infection, clinical characteristics, laboratory diagnosis and treatment measures, as well as prevention of monkeypox infection.

## Epidemiology of monkeypox

It was in 1970 that the first patient of human monkeypox was diagnosed in the DRC.^33^ After that, more than 400 monkeypox patients were documented in Africa between 1970 and 1990. Of these, the vast majority were diagnosed at DRC,^28,52–54^ followed by 6 cases in Central African Republic (CAR),^55^ 2 cases in Cameroon,^56,57^ 3 cases in Nigeria,^14,58,59^ 2 cases in Ivory Coast,^60^ 4 cases in Liberia,^58^ and 1 case each in Sierra Leone and Gabon.^56^ In 1990, there have been four cases of suspected monkeypox in Cameroon, one of which has been confirmed.^61,62^ In 1996, the DRC experienced a prolonged outbreak of human monkeypox. Initially, a patient was confirmed in February 1996, and until August of that year, a total of 71 suspected monkeypox cases were reported. As of 1999, over 500 patients of monkeypox were reported in the DRC.^61,63–65^ From February to August 2001, a total of 16 cases of monkeypox were identified in the province of Equateur in the DRC.^66^ Meanwhile, a study conducted by Anne et al.^67^ in the DRC from 2001 to 2004 examined vesicle fluids and crusted scabs from 136 people suspected of having monkeypox. They found 51 patients of monkeypox.

Human monkeypox attracted little attention worldwide until the first outbreak outside Africa occurred in the United States in 2003.^68,69^ Forty-seven cases, with confirmed (37 patients) and suspected (10 patients) monkeypox were reported, and all people infected by contact with pet prairie dogs (*Cynomys* spp.).^70^ The pets were infected after being in close proximity to imported small mammals (African rodents, rope squirrels (*Funiscuirus* spp.), tree squirrels (*Heliosciurus* spp.), Gambian giant rats (*Cricetomys* spp.), brushtail porcupines (*Atherurus* spp.), dormice (*Graphiurus* spp.), and striped mice (*Hybomys* spp.) from Ghana. According to CDC, laboratory tests using virus isolation and PCR amplification have proved that at least three dormice, two rope squirrels, and one Gambian giant rat were found to be infected by monkeypox. It was noted that no deaths occurred and no human-to-human transmission was observed,^68^ which was explained by the strain being a West African strain, based on genetic analysis.^69^ In addition, a study has conducted to evaluate the effects of the route of infection on the clinical illness of human monkeypox and demonstrated that significant symptoms of systemic diseases were more likely to occur in cases exposed to complex exposures than in cases exposed to noninvasive exposures (49.1 vs. 16.7%; *P* = 0.041).^70^ In contrast to Africa, a large number of cases confirmed in this outbreak are adults.^71^ In 2003, the Republic of Congo (ROC) reported the first outbreak of human monkeypox. It was recorded in this outbreak that 11 patients of monkeypox were confirmed and probable, all of whom were younger than 18 years of age, one death occurred. It is believed that the monkeypox virus has been transmitted from human to human six consecutive times, making it the longest recorded series of consecutive transmissions of monkeypox from one individual to another.^72^ In Unity State, Sudan, between September and December 2005, ten confirmed and nine possible patients of monkeypox were described in 5 villages (2 in Bentiu, 3 in Modin, 5 in Nuria, 5 in Rubkona, and 4 in Wang Kay).^73^ Between 2010 and 2018, varying numbers of monkeypox patients were documented in several African countries, including DRC,^74,75^ CAR,^76–78^ Cameroon,^79,80^ Liberia,^76^ Sierra Leone,^81^ as well as COA.^82^ In addition, it was reported in 2017 that Nigeria was experiencing an outbreak. 122 confirmed or possible patients of human monkeypox were reported between September 2017 and September 2018 in 17 states in Nigeria. Six deaths were recorded (case fatality rate 6%).^83–85^

Thousands of people around the world have been infected by the monkeypox outbreak in 2022, which followed several years of sporadic cases outside of Africa (United Kingdom,^86–88^ Singapore,^89,90^ Israel,^91,92^ United States^93^). On May 2022, multiple cases of monkeypox were identified in the United Kingdom.^2^ On 6 May 2022, an individual with travel ties to Nigeria was diagnosed with monkeypox.^94^ On 12 May, the UK Health Security Agency (UKHSA) in London confirmed two additional cases of monkeypox.^95^ There is no link between the two of them and they have never traveled to the endemic region. A week later, another four cases were confirmed. Unusually, there was no known contact between these patients and the previous confirmed cases.^96^ By polymerase chain reaction (PCR) experiment of patient swab samples, the outbreak was caused by the West African clade of monkeypox virus, which is less fatal than the other known monkeypox variant (CB, Clade I) after infection, with a case lethally rate of only 1%.^39,50,97,98^

Portugal, Canada, and Spain documented 14, 13, and 7 cases of monkeypox, respectively, on 18 May.^99–101^ It was also the same day that the United States identified its first monkeypox patient of 2022.^102^ On 19 May, Belgium and Sweden reported their first cases.^103,104^ Belgium announced that monkeypox cases were required to be isolated for 21 days at once. Belgium also became the first country in the world to require self-isolation of monkeypox cases. At the same time, Italy has reported first case, a traveler from the Canary Islands.^105^ Besides, France has reported a suspected case. Genomic analysis the virus isolated from a monkeypox virus infecting case in Portugal showed that the virus also belonged to the WA branch of evolution, closest to the virus carried by cases imported from Nigeria in 2018 and 2019.^43^ Two patients of monkeypox were confirmed in Australia on 20 May, both of whom had recently returned from travel to Europe.^106^ On the same day, the first cases were confirmed in Germany, as well as in the Netherlands and France.^106–110^ On the next day, the first cases were confirmed in both Switzerland^111^ and Israel,^112^ and the patient documented by the Israeli Ministry of Health is also the first case of monkeypox in Asia.^113^

After May 2022, a large number of monkeypox patients were confirmed in non-endemic countries worldwide. This unusual outbreak promoting the WHO to declare monkeypox as an “evolving threat of moderate public health concern” on June 23. Furthermore, the WHO announced that monkeypox outbreaks in many countries and regions constitute a “Public Health Emergency of International Concern” (PHEIC) on July 23, 2022.^114,115^ Meanwhile, monkeypox prevention and treatment guidelines have been issued in several countries around the world.^116^ As of 13 September 2022, 57,995 monkeypox virus infections which were laboratory confirmed have been reported in >100 countries or regions across all six WHO regions^13^ (Fig. 1 and Table 1). Of these, a total of 18 deaths were reported in 9 countries. In particular, a very recent news indicated that the first person of monkeypox was confirmed in Hong Kong on September 6.^117^ Epidemiological investigation revealed that this patient arrived in Hong Kong from the Philippines on September 5 at the age of 30 years. Consequently, as a result of the monkeypox emergence in Hong Kong, a preparedness and response plan has been activated by the Hong Kong Government. Also, this is the first reported person of monkeypox since the four cases were confirmed in Taiwan Province of China.^118,119^ Notably, the U.S. Centers for Disease Control and Prevention (CDC) declared monkeypox a public health emergency on August 4, 2022 in the United States.^120^ The monkeypox epidemic in the United States is extremely serious, as of September 6, monkeypox had since spread to every state, with a total of 20,733 cases.^121,122^

## Monkeypox virus reservoirs

Monkeypox is a zoonotic disease whose natural reservoir remains unknown.^123,124^ Several researches have conducted to determine the reservoir or natural hosts of monkeypox virus, Khodakevich et al.^125,126^ Have reported that antibodies to the virus were found in 2 of 18 squirrels tested. The monkeypox virus was extracted from a diseased squirrel, *Funisciurus anerythrus*, which was the first report of monkeypox virus being isolated from a wild animal. Other researches have also suggested that squirrels of the genera *Funisciurus* and *Heliosciurus* are related to the natural cycle of monkeypox virus in DRC,^127–130^ as well as Rodents of the genera *Cricetomys, Graphiurus*, elephant shrews of the genus *Petrodromus*.^127^ In March 2012, monkeypox virus was separated from a wild-living monkey (a sooty mangabey) by Radonic et al.^131^ Its whole-genome sequence showed that it had significant similarities with monkeypox viruses located in Western Africa. Patrono and colleagues^132^ described the frequent appearance of monkeypox virus in a wild-living chimpanzees (*Pan troglodytes verus*, hereafter chimpanzee) population from Taï National Park, Ivory Coast. A number of animal species, mostly rodents and non-human primates, have been confirmed to be susceptible to the virus after multiple investigations,^133,134^ as listed in Table 2.

**Table 2:** Animal species susceptible to infection with monkeypox virus

Order	Family	Species	Common name	Reference
Didelphimorphia	Didelphidae	*Monodelphis domestica*, *Didelphis marsupialis*	Gray short-tailed opossum, Southern opossum	^483^
Eulipotyphla	Erinaceidae	*Atelerix* spp.	African hedgehog	^483^
Lagomorpha	Leporidae	*Oryctolagus cuniculus*	White rabbit	^484^
Macroscelidea	Macroscelididae	*Petrodromus tetradactylus*	Four toed sengis (elephant shrews)	^127,133^
Pilosa	Myrmecophagidae	*Myrmecophaga tridactyla*	New World giant anteater	^485^
Rodentia	Chinchillidae	*Chinchilla lanigera*	Chinchilla	^483^
	Cricetidae	*Sigmodon hispidus*	Cotton rat	^486^
	Dipodidae	*Jaculus* spp.	Jerboa	^483^
	Gliridae	*Graphiurus* spp.	African dormouse	^483,487^
	Muridae	*Mus musculus*, *Mastomy natalensis*, *Oenomys hypoxanthus*, *Rattus norvegicus*	House mouse, Multimammate mouse, Rufus-nosed rat, Brown rat	^133,134,488–491^
	Nesomyidae	*Cricetomys* spp.	Giant pouched rat	^483,492^
	Sciuridae	*Cynomys ludovicanus*, *Funiscirus* spp*.*, *Heliosciurus gambianus*, *Protexerus stranger*, *Marmota monax*, *Marmota bobak*, *Spermophilus tridecemlineatus*, *Sciurus vulgaris*, *Xerus* sp., *Funisciurus lemniscatus*, *Funisciurus anerythrus*, *Funisciurus ilsabella*, *Funisciurus congicus*, *Heliosciurus gambianus*, *Heliosciurus rufobrachium*	Black-tailed priarie dog, Rope squirrel Sun squirrel, Forest giant squirrel, groundhog, Ground squirrel, 13-Lined ground squirrel, Red squirrel, Unstriped ground squirrels, Ribboned rope squirrel Thomas’s rope squirrel, Lady Burton’s rope squirrel, Gambian sun squirrel, Red-legged sun squirrel	^125,127,129,130,133,483,490,493–499^
	Hystricidae	*Atherurus africanus*	African brush-tailed porcupine	^490^
Primates	Callitrichidae	*Callithrix Jacchus*	Marmoset	^500^
	Ceropithicediae	*Colobus* spp., *Cercocebus atys*, *Cercocebus galeritus*, *Cercopithecus Ascanius*, *Cercopithecus mona*, *Cercopithecus aethiops*, *Cercopithecus nictitans*, *Cercopithecus petaurista*, *Cercopithecus neglectus*, *Cercopithecus pogonias*, *Cercopithecus hamlyni*, *Cercopithecus* spp., *Colobus badius* (now *Procolobus badius*), *Allenopithecus nigroviridis*, *Macaca mulatta*, *Macaca fascicularis*, *Semnopithecus* spp., *Colobus badius*	Colobuses, Sooty mangabey, Agile or Crested mangabey, Black-cheeked white-nosed monkey/Red-tailed monkey, Mona monkey, Grivet, Putty-nosed Monkey, Lesser Spot-nosed Monkey, De Brazza’s Monkey, Crowned Monkey, Hamlyn’s Monkey, Guenons, Red colobus monkey, Allen’s swamp monkey, Rhesus monkey, Crab-eating Macaque, Gray langur, Red Colobus	^125,130,131,490,498,499,501–504^
	Hominidae	*Gorilla* sp., *Pan troglodytes*, *Pongo* sp., *Homo sapiens*	Gorilla, Chimpanzee, Orangutan, Human	^76,485,490^
	Hylobatidae	*Hylobates lar*	Lar Gibbon	^505^
	Cebidae	*Saimiri sciureus*	squirrel monkey	^505^
	Lorisidae	*Perodicticus potto*	West African Potto	^490^
Carnivora	Procyonidae, Felidae	*Nasua nasua*, *Felis* spp.	South American Coati, Felis	^483,490,498^
Artiodactyla	Suidae	*Sus scrofa*	Eurasian Wild Pig	^127^

## Novel transmission patterns

**Fig. 2:**
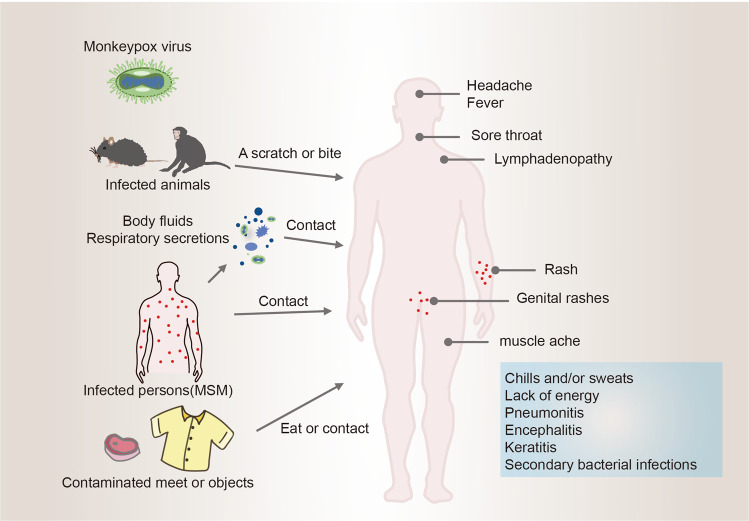
Schematic illustration of the transmission and clinical characteristics of monkeypox. There are many modes of transmission, animals infected with monkeypox virus (such as squirrels, rodents, monkey, and sooty mangabey), direct contact with body fluids or diseased parts of infected animals, scratching or biting by animals, consumption of meat from infected animals, sexual contact (MSM), and contact with contaminated objects, respiratory secretions from those infected individuals, skin lesions, along with their bedding and clothing. The clinical characteristics of monkeypox are depicted on the right side of the figure. General features such as lymphadenopathy, fever, headache, chills and/or sweats, sore throat, muscle ache, lack of energy, rash, and genital rashes are seen commonly. Complications of monkeypox include pneumonitis, encephalitis, keratitis, and secondary bacterial infections

Monkeypox transmission from animals to human may include direct contact with diseased parts or body fluids of infected animals, scratching or biting by animals, eating meat from infected animals, and contact with contaminated objects.^135–141^ Person to person transmission of monkeypox is caused by close contact with a human with monkeypox virus infection, including contact respiratory secretions from those infected individuals, skin lesions or genitals, prolonged face-to-face contact, along with their bedding and clothing^142–144^ (Fig. 2). There is very limited data on monkeypox infection during pregnancy, although a study has demonstrated the existence of vertical transmission of monkeypox virus.^145,146^ At the General Hospital of Kole, an observational research was conducted, Mbala et al. described the fetal outcomes for one of four pregnant women.^147^ Of the four women, one delivered a healthy baby, one had fetal death and two delivered miscarriages in their first trimester. In addition, monkeypox virus was detected in semen sample by researchers.^148^ Therefore, more attention is needed cause individuals such as children and pregnant women are more susceptible with increasing cases.^145,147,149–157^ Notably, a very recent report proved that human-to-dog transmission of monkeypox virus.^158^ Next-generation sequencing was used to compare the DNA sequences of the monkeypox virus from the dog and the patient. The samples both contained viruses of the human monkeypox virus-1 clade, lineage B.1, that have been spreading in countries that are not endemic to the disease.

According to previous reports, monkeypox virus was not previously been highly contagious. Monkeypox of the CB clade was found to have a lower basic reproduction number (R0) than 1 between 1980 and 1984 in the Democratic Republic of the Congo.^159,160^ Disseminated patients of monkeypox have been recorded in Africa, usually because of close contact with wild animals, especially rodents. Such travel related monkeypox individuals have limited secondary spread, making human-to-human transmission inefficient.^1,76,83^ However, a recent study by Du et al.^161^ reported that the reproduction number was estimated to be 1.39 (95% CrI: 1.37, 1.42) by aggregating all patients in 70 countries as of July 22 2022. According to initial estimates based on analysis of the first 255 PCR-identified patients of monkeypox in Italy in 2022, the reproduction number among men who have sex with men is 2.43 (95% CI 1.82–3.26).^162^ Which indicates that the disease has epidemic potential. Meanwhile, the vast majority of monkeypox patients had not recently traveled to the endemic regions of Africa, such as Nigeria, the DRC as well as central and western Africa.^163^ Human-to-human transmission of this virus is unusually frequent in this outbreak, suggesting close contact is the most likely route of transmission.^164^

Another unusual feature is that the large number of cases diagnosed with monkeypox are male, and a considerable part of patients have sex with men (MSM), particularly in Canada, Spain, and the UK, suggesting that sexuality is another forms of close contact, even though monkeypox was not considered a sexually transmitted disease before.^165–177^ It is reported that higher proportion of MSM infected is due to accidental entry into the community and then sexual behavior constituting “close contact” rather than sexual transmission of the monkeypox virus itself.^178^ However, a study of samples from 528 confirmed cases outside endemic areas in Africa between April and June showed that 98% of patients were bisexual men or gay and 95% of those infected were suspected of transmission through sexual activity.^179^ A prospective cohort study in Spain indicated that 91.7% cases identifying as MSM in 181 monkeypox infection cases.^180^ Besides, monkeypox virus DNA was detected by PCR in seminal fluid in 29 of the 32 cases.^179^ On 3 August, In the Monkeypox Surveillance Bulletin published by the ECDC-WHO Regional Office for Europe, 99% (15,439/15,572) of the cases were males, with 43.4% of MSM cases.^181^ Meanwhile, using a branching process transmission model, researchers showed that a small minority of people have disproportionately large numbers of partners, which could explain the continued increase in monkeypox patients among MSM population.^182^ Another research showed a transmission number of 2.43 in the MSM community among Italian cases in May-June 2022.^183^ According to Endo and colleagues,^182^ monkeypox’s basic reproductive number (R0) may be significantly higher than 1 over the MSM sexual contact network. Monkeypox virus spread model for assessing outbreak risk in a metropolitan area indicated that if transmission efficiency increases in the higher-risk group like gays and MSMs, broader populations may be affected.^184^ On the other hand, Bragezzi et al.^185^ conducted a meta-analysis to show that sexual contact is involved in 91% cases (total 124 cases). Notably, a recent study of 21,098 monkeypox cases (data from 41 countries, as of August 23, 2022) revealed that the vast majority were MSM, with a typical rash characteristic.^186^ Transmission was mainly through close contact during sexual activities. Overall, all these reports suggested that MSM was up to now the most frequently suspected route of transmission.^173,175,187–193^ Consequently, targeted interventions are needed in communities with a large part of MSM individuals to prevent further transmission. And further research is demanded to determine whether sexual transmission is possible.

## Monkeypox virus mutations and mechanisms of viral infection

Monkeypox virus is a species of double-stranded DNA virus which causes monkeypox in humans and other animals. It belongs to the genus Orthopoxvirus in the family Poxviridae. Among all animal viruses, poxviruses have the largest and most complex DNA genomes.^194,195^ There are four major elements of the virion: core, lateral bodies, outer membrane, and the outer lipoprotein envelope.^196^ The central core contains the viral dsDNA and core fibrils. The monkeypox virus genome consists of 197 kb with the central genomic region comprising of 101 kb.^17^ Both terminal variables regions include a 6379 bp terminal inverted repetition (ITR) with approximately 80 bp long hairpin loop, 70 or 54 bp short tandem repeats and unique ITR sequences NR 1 and NR 2 and the coding region.^32,197,198^ The virus contains about 190 nonoverlapping open reading frames (ORFs),^17,197,199,200^ four of which are located in the ITR sequence.^197^ Genes responsible for viral replication, transcription, assembly, and release are conservatively located in the genome’s central region, as they are in all orthopoxviruses.^200^ While most of the genes expressing virulence and host tropism are found at both ends of the genome. These terminal genes play a role in immune evasion by interfering with signaling, presentation, and recognition of antigens and apoptosis.^201–203^

Monkeypox virus has a low frequency of genomic mutations, due to the DNA double-stranded structure, and the 3’–5’ exonuclease activity of its DNA polymerase.^204,205^ However, the 2022 monkeypox virus diverges from the related 2018–2019 viruses by a mean of 50 single-nucleotide polymorphisms (SNPs), considering previous estimates of the substitution rate for orthopoxviruses, this is much higher than expected.^39,206^ Such a divergent branch could speed up the evolution of the monkeypox virus. Notably, further examination of the mutational profiles of these SNPs suggested a dramatic mutational bias, with 26 (14 non-synonymous, 10 synonymous, and 2 intergenic) and 15 (nine non-synonymous and 16 synonymous) being GA>AA and TC>TT nucleotide replacements, respectively. Several studies have indicated that mutations observed in viral genome editing may be caused by apolipoprotein B mRNA-editing catalytic polypeptide-like 3 (APOBEC3) enzymes.^207–212^ Another phylogenetic analysis indicated that the monkeypox virus‐2022 strains contained 46 new consensus mutations, including 24 nonsynonymous mutations, compared with the monkeypox virus-2018 strain.^213^ We suspected that the lower mortality and higher transmission of 2022 monkeypox virus than previous monkeypox virus may related to these viral mutations. Although these viruses are under continual adaptation, it is unknown whether these mutations contribute to help the virus evade host immunity. To further understand how these mutations affect viral function, additional research is required.

**Fig. 3:**
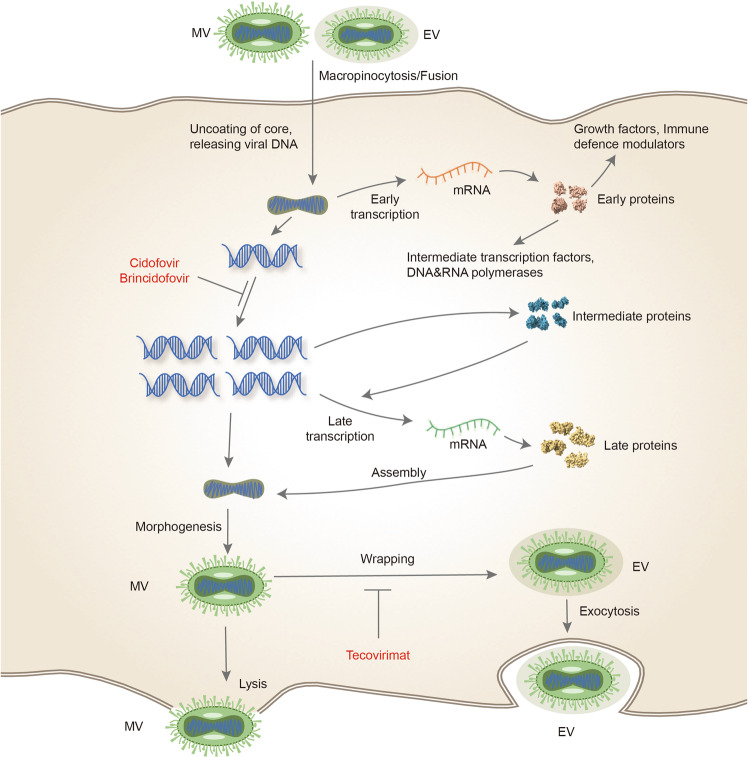
Monkeypox life cycle and mechanisms of action of antivirals. This diagram depicts the life cycle of monkeypox virus inside a human cell. Notably, Replication cycle of monkeypox virus occurs in the cytoplasm of the host cell. Following viral attachment, virion binds and fuses with the host cell membrane, the viral core is released into the cytoplasm of the host cell. Viral particles are assembled into intracellular mature viruses (MV), then stay in the cytoplasm as intracellular mature virions of released as extracellular enveloped viruses during cell lysis. MV can also wrap an additional envelope and attached to the cell membrane, then then release through exocytosis. Cidofovir and its prodrug brincidofovir inhibit the viral DNA polymerase during DNA replication. Tecovirimat targets the VP37 protein, which is vital for envelopment of intracellular mature virus with Golgi-derived membrane to form enveloped virus (EV), prevents the virus from leaving an infected cell, hindering the spread of the virus within the body

Unlike other DNA viruses, monkeypox virus replicates in the cytoplasm of the host cell.^196^ Poxviruses enter into the host cell may be divided into 3 steps (Fig. 3): virus attachment, hemifusion, and core entry, which occurs at the cell membrane or following endocytosis.^196,214,215^ A unique feature of poxviruses is that they produce two types of infectious particles: extracellular virions (EVs) and mature virions (MVs), both viral particles have different viral surface epitopes.^216^ Poxviruses enter cells through different mechanisms depending on their infectious forms, mature virion (MV) with single outer membrane, or enveloped virion (EV) with additional membrane and different protein composition.^217–222^

Despite not being able to identify specific receptors for poxviruses, many glycosaminoglycans including heparin sulfates and chondroitin, as well as laminin, contribute to their attachment to cells.^217,223,224^ Up to now, many researches have been demonstrated that several vaccinia virus proteins are vital for binding to the cell surface, including D8L,^225,226^ A27L,^227,228^ A34R,^229–232^ A26L^224^ and H3L.^233–235^ It is following viral attachment that the virion binds to the membrane and fuses with the host cell, causing its core to be released into the cell’s cytoplasm. Virion core contains enzymes and factors that initiate transcription. It was proven that L1, F9, A28, A27, L5, H2 were essential viral envelope proteins for cellular entry.^236–243^ Virus-encoded multi-subunit DNA-dependent RNA polymerase initiates viral transcription, which is followed by host ribosome-mediated translation of early, intermediate, and late proteins. The intermediate and late stages of viral gene transcription require cooperation with host-derived transcription factors.^244–248^ Synthesis of poxvirus DNA occurs in the cytoplasm. A majority of viral particles remain in the cytoplasm as intracellular mature virions (IMVs) and are encased within the protein matrix of scabs. A second envelope (intracellular enveloped virions, IEVs) may be acquired by the remaining virions, and they are attached to the cell membrane of their host.^249^ As cell surface-associated enveloped virions (CEVs), they cause cell-to-cell transmission of the virus, while extracellular enveloped virions (EEVs) contribute to systemic transmission.^250^

## Clinical characteristics

The clinical characteristics of monkeypox are quite similar to those of smallpox, but the monkeypox is clinically milder.^37,251–253^ It is crucial to realize that monkeypox and smallpox differ mainly by the occurrence of lymphadenopathy,^141^ since 90% of monkeypox patients suffer from lymph node enlargement.^254^ Monkeypox is most commonly confused with chickenpox, caused by varicella-zoster virus (VZV). In terms of their clinical characteristics, these two diseases share a great deal in common. Moreover, several studies have reported that individuals are infected both monkeypox virus and VZV.^66,67,127^

The incubation time of monkeypox virus is usually 5–21 days.^162,183,255,256^ According to Miura’s report, incubation period (18 cases) is estimated to be 8.5 days on average.^255^ Based on 22 possible (*N* = 1) and confirmed (*N* = 21) monkeypox patients reported in the United States through 6, 2022, Charniga and colleagues estimated the incubation time for monkeypox.^256^ According to their study, the incubation time from exposure to the onset of symptoms was 7.6 days on average. Besides, another research found that among 23 people with known history of exposure, the median incubation period was 7 days (between 3 and 20 days).^179^

In humans, monkeypox disease can be divided into two phases: the prodrome and the rash. Initial symptoms of monkeypox virus infection include headache, lack of energy, fever, chills and/or sweats, sore throat, muscle ache, and lymphadenopathy^5,257–260^ (Fig. 2). Usually, few days after fever and lymphadenopathy, the rash appears. Rash starts with the face and then appears the whole body, and is characterized by a few to several thousand lesions.^83,91,261^ As the rash progresses about 2–4 weeks, plaque is replaced by papules, blisters, pustules, scabs, followed by shedding.^28,254,262^ Complications of monkeypox include encephalitis, keratitis, pneumonitis, and secondary bacterial infections.^28,143,254,263^

In the United States, a report on the epidemiological and clinical features of monkeypox cases showed 42% of persons (*n* = 291 participants) has no prodromal symptoms, and 37% of patients did not have fever as of the time of assessment, despite most patients included typical features.^122^ And genital rashes were observed in many cases, which was also recently proven in another studies.^264–270^ Other recent reports describe similar clinical characteristics.^180,271,272^ Patel and colleagues^272^ demonstrated that all 197 monkeypox infection cases developed mucocutaneous lesions, typically in the genitals (111 of 197 patients) or the perianal areas, causing rectal pain and penile edema. Moreover, according to a case report from Spain, 78 of 181 persons had lesions in the oral and perioral region and 141 in the anogenital region. The following complications occurred in 70 of the 181 patients: 45 (24.9%) of 181 had a proctitis, 19 (10.5%) tonsillitis, and 6 (3.3%) had a bacterial skin abscess.^180^ The clinician should remain vigilant for monkeypox-like rashes regardless of whether the rash has disseminated or had a prodrome prior to the rash. Notably, the possibility of some monkeypox virus infections being asymptomatic has been raised by two recent case reports from Europe. It has been shown in several studies that monkeypox could go unobserved when it is propagable, so physicians and persons at high risk should be aware of this fact.^171,172,273^

Available data from the European Union, the United Kingdom, and the United States indicated that 28–51% have human immunodeficiency virus (HIV) infection among MSM patients with monkeypox.^122,167,179,180,272,274^ Currently, the risk for monkeypox infection is unknown, however, in people with HIV.

Symptoms of monkeypox last 2–4 weeks, and the disease is usually self-limiting. The lethality of monkeypox patients after infection depend on the clade of virus infected, route of infection, patient age, and patients infected with the CA clade of the virus is generally higher than the WA clade.^6,70,141,275^ Previous reports^80,83,97,276–278^ indicated that the case fatality rates (CFR) of monkeypox ranging from 1 to 11%. What’s more, in a systematic review that exclusively evaluated the case fatality rates (CFR) by clades, the overall rate of case fatalities was confirmed to be 8.7%, which was significantly lower for West African clade (3.6%, 95% CI 1.7–6.8%) than Central African clade (10.6%, 8.4–13.3%).^1,66^ Among 1958 patients, some 4% (95% CI 1–9%) of hospitalized patients had fatal outcomes.^260^ In the current 2022 monkeypox outbreak, it was reported that patients at least sixteen years old always have milder symptoms in the current investigations, with a pooled case fatality rate of 0.03% (1 of 2941 patients).^279^ Monkeypox is more often fatal in children, which is similar to smallpox.^36,80^ As of September 2022, a total of 15 deaths have been documented in this monkeypox global outbreak, in which 9 deaths in locations that have historically recorded monkeypox.

## Laboratory diagnosis

Diagnosis of monkeypox requires a combination of clinical symptoms, epidemiological information, and laboratory tests. According to WHO recommendations, nucleic acid amplification testing (NAAT) has been conducted for monkeypox virus detection.^280^ Genes commonly used for conventional polymerase chain reaction (PCR) testing include hemagglutinin,^281^ the acidophilic-type inclusion body gene,^282,283^ and the crmB gene.^284^ Compared with other diagnostic methods, real-time PCR provides high throughput, high sensitivity, and fast results. Several genes have been developed to detect monkeypox virus, such as envelope protein gene (B6R),^285^ B7R gene,^286^ DNA polymerase gene E9L,^69,285^ complement binding protein C3L,^278,287^ DNA-dependent RNA polymerase subunit 18 (RPO18) gene,^288^ F3L and N3R.^289^ Meanwhile, recombinase polymerase amplification (RPA) method has been proven to be an alternative to real-time PCR. It was reported that the monkeypox virus-RPA-method was especially sensitive with a limit of detection (LOD) of 16 DNA molecules per microliter by targeting the tumor necrosis factor binding protein gene.^290^ With different target sites in the viral genome, these tests have varying sensitivities and limits of detection for monkeypox virus.^291^ According to Li et al.^278^ real-time PCR assay for generic monkeypox virus has a limit of detection for 0.7 fg (∼3.5 genomes), while monkeypox virus West Africa specific (G2R_WA) assay’s LOD is at 1.7 fg (∼8.2 genome), and monkeypox virus Congo Basin (C3L) assay’s LOD is at 9.46 fg (∼40.4 genomes). As well, Maksyutov et al.^292^ developed real-time PCR assay that targets viral F3L gene with a LOD of 20 copies per reaction. Several other studies reported LOD for monkeypox virus assays of 6.359 copies/mL,^293^ 20 copies/reaction,^286^ 11–55 fg (50–250 copies of each gene),^289^ 2 fg (~10 viral copies),^285^ 0.05 fg (25 copies/assay).^294^

Recently, a high-throughput molecular testing method for monkeypox virus was established,^293^ which may expand the detection capacity and reduces the detection of time. In the current outbreak, the whole genome sequencing was also used to identify the monkeypox strain.^40,92,198,295^ What’s more, there are several other DNA-based assays have been investigated for monkeypox virus detection, such as loop-mediated isothermal amplification (LAMP),^296^ restriction length fragment polymorphism (RFLP),^98,297^ as well as recombinase polymerase amplification (RPA).^290^

Laboratory methods for detecting monkeypox also include enzyme-linked immunosorbent assay (ELISA),^298,299^ western blot (WB)^136,300^ and immunohistochemistry (IHC).^30,301^ It is often necessary to use serologic diagnostic procedures to diagnose poxvirus when there is no virological sample. In most cases, ELISA is the most frequently used serologic test. Serological testing of the specific IgM and IgG antibodies are commonly deployed. As soon as the rash appears, IgM antibodies appear and rise approximately 2 weeks before declining and disappearing within 1 year. While IgG antibodies also generate quickly after the rash onset, rise approximately 6 weeks, and lasts for decades.^302^ However, the specificity is low, due to the immunologic cross-reactivity between monkeypox virus and other othopoxviruses.^37,251^ Electron microscopic observation can be used as an auxiliary method to detect monkeypox virus.^24,303^ However, due to the laborious and costly sample preparation, it is difficult to be used popularly.

## Treatment

Treatment is suggested for monkeypox virus-infected individuals with severe disease at present or who may be at high risk for developing serious illness (people who are immunocompromised, pediatric population, have characteristic dermatitis or history, have skin symptoms of exfoliation, pregnant or breastfeeding woman, one or more complications), or those with monkeypox virus aberrant infections, including accidental implantation in mouth, eyes, or other anatomical areas where monkeypox virus infection that could pose a special threat (such as the genitals or anus). For most patients, treatment is symptomatic and supportive. Despite the fact that monkeypox has no specific treatment, smallpox antiviral drugs such as brincidofovir, tecovirimat, and cidofovir may have effect against monkeypox because of their similar genetics.^258,304–308^

### Tecovirimat

Tecovirimat (also known as TPOXX, ST-246), a small-molecule inhibitor of virus, is effective against orthopoxviruses both in vitro and in vivo, including vaccinia virus, camelpox virus, cowpox virus, mousepox virus, variola viruses, and monkeypox virus.^309^ Tecovirimat targets the VP37 protein, inhibits the spread of viruses within the body by preventing them from leaving infected cells,^309,310^ as shown in Fig. 3. Tecovirimat inhibits neither DNA or protein synthesis nor the formation of mature virus. The mature virus remains in the host cell until cell lysis. Tecovirimat displayed s strong antiviral efficacy with an EC50 range of 0.01 to 0.07 μM.^309,311,312^ Several studies have demonstrated that the VP37 protein plays an essential role in encapsulating intracellular mature virus with Golgi-derived membrane to form enveloped virus.^309,313–317^

Tecovirimat has a distinguished antiviral effect against the monkeypox virus lineage responsible for the 2022 outbreak in vitro.^318^ Further, the effectiveness and safety of tecovirimat has been revealed in multiple animal studies.^319–324^ Treatment with 10 mg per kilogram of body weight of tecovirimat for 14 days in the monkeypox model could achieve >90% survival.^324^ In a nonhuman primate (NHP) model, by taking orally once a day, tecovirimat was found to be effective in protecting NHP from monkeypox virus illness, with reduced viral loads, fewer rashes, prolonged survival and significantly reduced mortality in the treatment group compared with the vehicle mongkeys.^325^ Smith et al.^326^ assessed the efficacy of tecovirimat against monkeypox virus challenge of 65 times the 50% lethal dose (LD_50_) in prairie dogs, and found that one hundred percent of animals that received tecovirimat survived challenge, while 75% of infected animals receiving vehicle alone succumbed to infection. Meanwhile, multiple phase 3 clinical trials of tecovirimat have demonstrated its safety and well tolerated (Table 3).

**Table 3:** Treatment and prevention of orthopoxviruses

Categories	Name	Mechanism of action	Administration	Typical dosage	FDA approval status	Clinical trials^a^	Reference
Antiviral medicine	Tecovirimat	Tecovirimat targets the VP37 protein, inhibits the spread of viruses within the body by preventing them from leaving infected cells	PO, IV	Oral dosage by body weight: 13 kg to less than 25 kg: 200 mg (1 capsule) every 12 h 25 kg to less than 40 kg: 400 mg (2 capsules) every 12 h 40 kg to less than 120 kg: 600 mg (3 capsules) every 12 h 120 kg and above: 600 mg (3 capsules) every 8 h Duration of therapy: 14 days Intravenous infusion dosage by body weight: 3 kg to less than 35 kg: 6 mg/kg every 12 h by IV infusion over 6 h 35 kg to less than 120 kg: 200 mg every 12 h by IV infusion over 6 h 120 kg and above: 300 mg every 12 h by IV infusion over 6 h Duration: up to 14 days	Smallpox, 2018	NCT05380752, NCT02080767, NCT02474589, NCT03972111, NCT00907803, NCT04392739, NCT04485039, NCT04971109, NCT04957485, NCT00728689	^324^
	Cidofovir	Prevent viral replication and transcription by inhibiting viral DNA polymerase	IV	5 mg/kg via IV infusion once	CMV retinitis in patients with AIDS, 1996	NA	NA
	Brincidofovir	Inhibits the synthesis of viral DNA by inhibiting the DNA polymerase activity or by acting as an acyclic nucleotide and binding to the viral DNA strand	PO	Adults and children weighing 48 kilograms (kg) or more—200 milligrams (mg) (two 100 mg tablets) once a week for 2 doses (on Days 1 and 8). Children weighing less than 48 kg—Use of oral liquid is recommended	Smallpox, 2021	NCT01143181,	^386^
Blood product	Vaccinia immune globulin	Antibody isolated from healthy donors previously vaccinated and inhibit viral infection	IV	Administered at a dose of 6000 U/kg	Treatment of complications resulting in smallpox vaccination including eczema vaccinatum, 2005	NA	NA
Vaccine	ACAM2000	Second generation, a live attenuated vaccinia vaccine	TDD	Reconstituted by addition of 0.3 mL of diluent to the vial containing lyophilized vaccine	Smallpox, 2007	NCT01158157, NCT01913353, NCT02977715, NCT00082446, NCT02038881, NCT00857493, NCT01144637, NCT00316524, NCT03699124, NCT00437021, NCT00133575, NCT00607243	^424,445,506–513^
	JYNNEOS	Third generation, highly attenuated vaccinia virus	SC	Administer two doses (0.5 mL each) of JYNNEOS 4 weeks apart	Smallpox and monkeypox, 2019	NCT00914732, NCT02977715, NCT01144637, NCT00316524, NCT00437021, NCT00565929, NCT03699124, NCT02038881, NCT01144637, NCT00316524, NCT00565929, NCT00133575, NCT02977715	^424,425,452,507,509–512,514^
	LC16	A live, replicating attenuated third-generation vaccines	ID	10^8^ plaque-forming units/mL, 0.02 μL	Smallpox, 1975	NCT00103584	^465^

*NA* not available

^a^Clinical phase 3 and published clinical trials are shown

In 2018, the FDA approved its first use for treating smallpox after studies showed it was safe in humans and effective in animals with similar viruses.^304,323,327,328^ A trial in monkeypox people showed the tecovirimat (600 mg twice daily for 2 weeks orally) did not cause side effects and that viral shedding and illness lasted shorter time.^329^ Besides, data collected from 25 cases with diagnosed monkeypox infection had completed a course of tecovirimat therapy revealed all cases tolerated the antiviral medicine well, with minor side effects. The lesions and pain of 10 patients (40%) were completely resolved by day 7 of therapy, and 23 of 25 patients on day 21. The most frequently adverse events of therapy reported including diarrhea in 2 (8%), itching in 2 (8%), nausea in 4 (16%), headache in 5 (20%), and fatigue in 7 patients (28%).^308^ Moreover, several reports have also documented the efficacy and safety of tecovirimat in the treatment of monkeypox.^308,330–332^ Healthcare providers can now provide tecovirimat treatment to monkeypox patients under an expanded access Investigational New Drug (EA-IND) protocol developed by CDC and FDA. A total of 1001 cases receiving this antiviral drug have been documented, in addition, data was abstracted by the Centers for Disease Control.^333^

Despite this, more clinical trials are needed to determine whether this antiviral drug is effective and safe for treating monkeypox infections in people.^334^ A double-blind, randomized controlled study will be conducted by the National Institutes of Health’s (NIH) National Institute of Allergy and Infectious Disease (NIAID) in treating individuals confirmed with monkeypox in adults and children to evaluate the effectiveness and safety of tecovirimat.^335^ Meanwhile, through the AIDS Clinical Trials Group, NIH/NIAID is conducting a phase 3 double-blind, randomized controlled investigation of tecovirimat in outpatient in the United States settings to treat monkeypox.^335^ According to the latest report, it is currently underway or planned to conduct several clinical experiments to evaluate tecovirimat’s safety and effectiveness in treating monkeypox patients (PALM-007, PLATINUM, WHO/ARNS, and ACTG5418).^336,337^

### Cidofovir

Since 1996, Cidofovir (CDV, also known as Vistide) has been approved for clinical use to treat cytomegalovirus (CMV) retinitis in persons with acquired immunodeficiency syndrome (AIDS).^338–341^ CDV is a prodrug, and it must be phosphorylated by enzymes of the cytoplasm after enter the host cells, and into CDV diphosphate (CDV-pp), which has a prolonged half-life.^342–344^ CDV-pp inhibits the viral DNA polymerase during DNA replication, as well as inhibits DNA polymerase 3′–5′ exonuclease activity.^343^

Many animal models and experiments in vitro have reported the effectiveness of cidofovir for treatment orthopoxviruses, including vaccinia, mousepox, as well as monkeypox.^338,345–350^ Between 72 to 96 h post-infection, treatment of mice inoculated with cowpox virus (CV) or vaccinia virus (VV) resulted in significant protection. And when given at least 5 days before infection or 3 days after infection with either VV or CV, a single-dose pretreatment or posttreatment with CDV at 3 to 100 mg/kg was effective.^351^ Another mouse model demonstrated that it was completely protective against virus-induced cutaneous lesions and against the associated mortality when started on the day of infection or the first post-infection day of topical treatment with cidofovir. In addition, systemic treatment with cidofovir caused lesions to heal and regress.^346^ The treatment of mice with cidofovir (for two days after 24 h of virus exposure) suppressed viral loads as well as cytokine levels in plasma and tissue, including interleukin (IL)-10, IL-6, IL-3, and IL-2.^352^ In non-human primate model, researches proved a significant reduction in mortality and cutaneous monkeypox lesions when CDV treatment is initiated 24 h after lethal intratracheal monkeypox virus infection.^353^

Various studies have shown the effectiveness of CDV against poxvirus. Patients with recalcitrant molluscum contagiosum virus (MCV) treated with intravenous cidofovir exhibited dramatic cleaning of their MCV lesions, and all three cases remain clean of recurrence.^354^ The use of topical CDV was found to be effective in treating molluscum contagiosum (MC) in a 12-year-old boy with Wiskott–Aldrich syndrome.^355^ Two children with AIDS were also successfully treated with topical 3% cidofovir in a combination vehicle (Dermovan) for recalcitrant MC.^356^ CDV has also been documented to be effective against cowpox and human vaccinia in additional case reports.^357–359^ Despite the lack of clinical evidence to support its use in treating monkeypox, cidofovir is generally thought to be effective. According to the CDC’s guidance, an outbreak of orthopoxviruses, including monkeypox virus, can be treated with this anti-viral medicine.

### Brincidofovir

Brincidofovir (also known as CMX001 or Tembexa) is a lipid conjugate of the acyclic nucleotide phosphonate, cidofovir (CDV). After brincidofovir entry into target cells, a phospholipase enzyme cleaves the lipid ester linkage in brincidofovir, liberating CDV and activated by two sequential phosphorylations, first yield cidofovir monophosphate (CDV-P) and then yield cidofovir diphosphate (CDV-PP).^360^ CDV-pp suppresses the synthesis of viral DNA by inhibiting the DNA polymerase activity of several double-stranded DNA (dsDNA) viruses or by acting as an acyclic nucleotide and binding to the viral DNA strand^361–365^ (Fig. 3).

Brincidofovir has under gone numerous animal studies^328,366–374^ to demonstrate its antiviral efficacy against double-stranded DNA viruses including poxviruses such as monkeypox virus and is currently in human clinical investigations for treating other double-stranded DNA viruses (Table 3). In addition, An antiviral regimen with brincidofovir and ST-246 administered on the day of monkeypox virus infection protects mice with the STAT1-deficient C57BL/6 mouse model.^375^ Lethal rabbitpox models revealed that brincidofovir administration at or before the midpoint of illness could reduce the fatality rate from rabbitpox virus infection, as well as reduce viral loads, which might contribute to decrease infectivity of the virus.^376,377^ Studies have been conducted using the prairie dog monkeypox virus model to determine the effectiveness of anti-poxvirus therapeutics.^378,379^ In a monkeypox virus animal model, brincidofovir was administered orally to prairie dogs to determine its pharmacokinetics (PK). It was found that BCV treatment early in the illness tended to increase survival against a lethal monkeypox virus challenge; the earlier the treatment was initiated, the greater the chance of survival.^380,381^ In summary, these models suggest that treating monkeypox patients early with brincidofovir is more likely to have better outcomes.

In 2021, Brincidofovir was licensed by the FDA for treating human smallpox disease in both adults and children. To facilitate the use of brincidofovir for the treatment of monkeypox, the CDC is currently developing an Expanded Access Investigational New Drug.^382^ In comparison with cidofovir, brincidofovir has higher intracellular levels of the active drug, well oral bioavailability, superior antiadenoviral activity, and no nephrotoxicity.^314,375,383–387^ The complete resolution in a patient with progressive vaccinia (PV) demonstrated the effectiveness of brincidofovir in combination with other antiviral drugs.^388^ Recent research, however, indicates brincidofovir is associated with serious adverse effects in monkeypox patients. Over the past three years, three cases have experienced liver toxicity in the United Kingdom.^329^ Further studies are warranted in humans regarding the efficacy of brincidofovir against monkeypox virus.

## VIGIV

Vaccinia immune globulin intravenous (VIGIV), a FDA-approved medicine, is applied to treat smallpox vaccination complications such as severe generalized vaccinia, vaccinia infections in people who have skin conditions, eczema vaccinatum, progressive vaccinia and aberrant infections induced by vaccinia virus (excluding isolated keratitis).^389^ Vaccinia immune globulin (VIG) is a sterile solution made up of high titers of IgG antibodies against the vaccinia virus taken from healthy persons who had been previously vaccinated against the live vaccinia virus.^390^ CDC allows the use of VIG for the treatment of monkeypox in an outbreak. Several studies have reported the patients received VIGIV for the treatment of orthopoxvirus infection.^391–393^ Nevertheless, data on VIG’s effectiveness in treating monkeypox virus infection are unavailable. Physicians may consider using it in severe cases. The VIG is also suitable for prophylactic use in persons with severe immunodeficiency in T cell function for whom smallpox vaccination is contraindicated after exposure to monkeypox virus.

## Monkeypox prevention

To prevent getting monkeypox, CDC suggest that people need to avoid close, skin-to-skin contact with someone who has monkeypox-like rashes, avoid contact with objects and materials that a case with monkeypox has used, use hand sanitizers that contain alcohol before eating, touching the face, and wash your hands frequently after using the bathroom.

There is no vaccine specifically designed to prevent monkeypox virus infection. Owning to immunological cross protection among orthopoxviruses,^194,298,394–398^ smallpox vaccines (vaccinia virus-based) were recommended for use in the current outbreak of monkeypox.^279,399–405^ Epidemiological data on human monkeypox collected in Zaire from 1980 to 1984 revealed that there was a significant difference in attack rates between contacts without a vaccination scar and those who had been vaccinated in the past (7.2% vs. 0.9%).^406^ Rimoin et al. suggested that monkeypox incidence increased dramatically in the DRC 30 years after smallpox vaccination campaigns ended.^407^ Furthermore, their data suggested that vaccine-induced immunity lasts a long time as individuals vaccinated against smallpox >25 years ago are still at reduced risk of monkeypox today. As demonstrated by the US monkeypox outbreak of 2003, smallpox vaccination can potentially sustain cross-immune protection against West African monkeypox for decades afterward. Three individuals who had not previously been infected with monkeypox virus and had been vaccinated against smallpox for decades were unaware of their subsequent infection because none of them had any clinical characteristics associated with the monkeypox.^298^

**Fig. 4:**
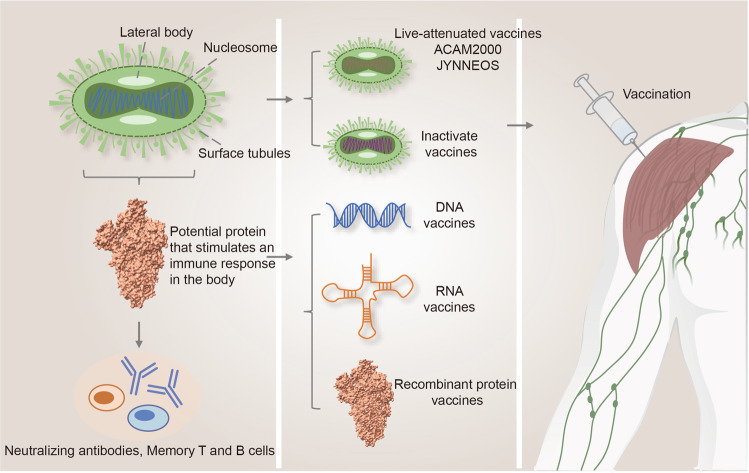
Development of monkeypox vaccines in the future. Currently, live attenuated vaccines such as ACAM2000 and JYNNEOS are clinically recommended for the prevention of monkeypox, but these vaccines were originally designed to prevent smallpox. Therefore, there is a demand for the development of vaccines that specifically prevent monkeypox, such as inactivated vaccines, DNA vaccines, RNA vaccines, and recombinant protein vaccines. These types of vaccines have proven their efficacy and safety in the COVID-19 epidemic

Studies have reported that first-generation live vaccinia vaccines can provide approximately 85% protection against monkeypox infection due to cross-reactive antibodies that can be induced by orthopoxviruses.^160^ Second-generation vaccine is ACAM2000, a live attenuated vaccinia vaccine approved in the USA in August 2007 for prevention of smallpox^408,409^ (Fig. 4). Its effectiveness has been demonstrated in both animal models^327,410–412^ and clinical trials (Table 3).

Assessment of the protective effect against aerosolized monkeypox virus in cynomolgus macaques revealed that a single immunization with ACAM2000 vaccines were protected completely.^410^ Monkeys challenged with lethal monkeypox virus after vaccinated with the ACAM200 live attenuated smallpox vaccine, all (*N* = 12) vaccinated treated monkeys survived.^412^ In a prairie dog model, for ACAM2000, the vaccine administered at 1 day postexposure was similarly effective with the vaccine administered at 3 days postexposure, resulting in 50–62% survival, compared with only 25% survival in the unvaccinated group.^411^ Taken together, ACAM2000 is likely effective at preventing monkeypox; however, due to its potential for replication, it may cause severe side effects, including progressive vaccinia, eczema vaccinatum, and myopericarditis.^388,409,413–421^ A person with a weak immune system, including someone with HIV, pregnant women, and those who suffer from skin conditions like eczema, are not advised to use it.^150,422^

The third-generation vaccine, modified vaccinia Ankara (MVA, JYNNEOS in the United States, IMVANEX in the European Union, and IMAMUNE in Canada), was approved in individuals 18 years of age or older considered to be more susceptible for smallpox or monkeypox infection in US to prevent both smallpox and monkeypox disease in 2019.^423^ Unlike ACAM2000, JYNNEOS is a nonreplicating vaccine, which will not lead to the production of live virus. As a smallpox vaccine, JYNNEOS has demonstrated good immunogenicity, safety, and efficacy both in animal models and clinicals.^424–436^ It was the only currently FDA-approved vaccinia vaccine for the prevention of monkeypox disease^437^ (Fig. 4). Several animal model studies exhibited good protective effects against monkeypox,^411,438–440^ furthermore, in macaque challenge trials 100% protection against monkeypox infection was demonstrated, along with long lasting immunity^410,441^ and long lasting protective immunity for a lethal monkeypox challenge.^442^ Petersen et al.^443^ evaluated its effectiveness, immunogenicity, and safety in healthcare workers at risk of monkeypox virus infection in the DRC. Meanwhile, another human clinical investigation examining the effectiveness of monkeypox vaccination after exposure is currently underway.^444^ Numerous clinical trials of MVA^445^ have proved the efficacy and safety of JYNNEOS, as listed in Table 3.

As a result of the outbreak of monkeypox in 2022, several countries, including the United States, Spain, Germany and the United Kingdom, have announced they are buying vaccines and/or releasing vaccines them from national stockpiles to combat the epidemic. JYNNEOS is being used by the US for pre-exposure vaccination of people who are at risk of occupational exposure to orthopoxviruses according to CDC announcements made in May 2022.^399,446^ Meanwhile, in its interim guidance for monkeypox vaccines, WHO recommended post-exposure prophylaxis (PEP) ideally within 4 days of the first exposure, pre-exposure prophylaxis (PrEP) is recommended for health workers at risk, clinical laboratory staff performing diagnostic testing, laboratory personnel working with orthopoxviruses, and others at risk under national policy.^447^ IMAMUNE is approved for immunization against monkeypox and Orthopox virus by the Public Health Agency of Canada (PHAC), and IMVANEX is recommended by the European Medicine Agency (EMA) for monkeypox prevention.^448,449^ By July 27, 786,000 more doses are expected to be released that week after the federal government distributed 300,000 doses of vaccine to state and local health authorities and clinics throughout the country. JYNNEOS is manufactured exclusively by Bavarian Nordic, which has a limited production capacity. Recently, due to the vaccine shortage, Bavarian Nordic has expanded its capacity by working with U.S. contract manufacturer to fill smallpox/monkeypox vaccines.^450^ In addition, a European Union Authorization (EUA) was approved by the FDA for the JYNNEOS vaccine, allowing healthcare providers to administer it intradermally to individuals 18 years of age and older who have been considered to be more susceptible for monkeypox infection.^451^ Individuals vaccinated intradermally were less vaccinated (by one-fifth) than those vaccinated subcutaneously (SC). The total dose available for use could increase by five-fold as a result. A study with lower intradermal doses of MVA was found to be immunologically noninferior to the standard subcutaneous dose (NCT00914732).^452^

In 1975, the other smallpox vaccine, LC16m8, was licensed for active immunization against smallpox in Japan. This vaccine is derived from the Lister (Elstree) strain of vaccinia and is a live, replicating, attenuated 3rd generation vaccine.^453^ This high attenuation resulted from a single nucleotide deletion mutation in the B5R viral gene, resulting in a truncated membrane protein B5, which is one of the most immunogenic proteins.^454–456^ Several animal model challenge studies of safety and efficacy of LC16m8 have been conducted in mouse,^454,457–461^ rabbit,^459,460^ and nonhuman primates.^462,463^ Both the intranasal and subcutaneous inoculation models showed no symptoms of monkeypox after immunization with LC16m8.^462^ A single vaccination of non-human primate model revealed that LC16m8 exhibited long-lasting protection against monkeypox virus, evidence from the reduction in viremia and the IgG antibody response.^464^ Notably, another nonhuman primate model revealed that for immunocompromised individuals, LC16m8 is a safer and more effective alternative to ACAM2000 and Dryvax (first-generation vaccines).^463^ LC16m8 was shown to produce neutralizing antibodies for vaccinia, monkeypox, and variola major, as well as broad T cell responses in a phase I/II clinical trial.^465^ In addition, various immunogenic studies in human have confirmed a good safety profile about LC16m8.^466–472^ In summary, these results reveal that LC16m8 may be effective for preventing people against monkeypox. Japanese authorities expanded the range of indications for this vaccine to include protection against monkeypox in August 2022.^473^

## Conclusion and perspectives

Monkeypox, COVID-19, and polio are among the three emergencies that WHO has declared worldwide at the moment. Monkeypox is now an international problem after previously being endemic to Africa. Until September 2022, more than 56000 patients have been diagnosed in the monkeypox 2022 outbreak. Monkeypox infection patients are rapidly increasing around the world, it is likely to be caused by a combination of natural and human factors. On the other hand, there has been an increase in human–wildlife contact due to climate change, deforestation, and the Ukrainian–Russian war, among other factors. In addition, it is still possible that monkeypox virus will become more prevalent due to the cessation of smallpox vaccination with vaccinia vaccine in 1980. Currently, in spite of the fact that mass vaccination is not recommended or achievable, individuals manage monkeypox may though vaccine (post-exposure prophylaxis, or pre-exposure prophylaxis), anti-viral medicines, as well as isolate or quarantine of the patients and any contacts with the patients.

Particularly, there are large number of patients have been confirmed in MSM population in this 2022 outbreak and has been related to unanticipated anal and genital lesions, suggesting the monkeypox virus may be spreading via sexual transmission.^158,170,179,180,185,272^ This novel transmission pattern was suspected during 2017 outbreak of human monkeypox in Nigeria, but was not proven.^474^ Meanwhile, MSM population has higher risk on other disease through sexual transmission, such as HIV. In Ogoina and colleagues’ study,^475^ it was reported that monkeypox patients in Nigeria who were HIV-positive had longer-lasting illness, and larger lesions, and a higher percentage of and genital ulcers and secondary bacterial skin infections. By contrast, Tarin-Vicente and his colleagues^180^ reported that there was no difference between HIV-positive patients and the rest of the population in terms of severity or progression of the disease. Therefore, more investigations are needed to understand the effectiveness of anti-viral treatment among individuals infected with both HIV and human monkeypox virus. Besides, in order to reduce monkeypox transmission within MSM groups, public health efforts must deal with challenges including homophobia, stigma, and discrimination.

Given the current global monkeypox epidemic, a race to create monkeypox -specific vaccines may occur like COVID-19 (Fig. 4). Moderna has announced that they have initiated a program to consider whether developing an mRNA monkeypox vaccine or not because of growing demand for vaccination, even though no further information on the vaccine or potential development timeline was released latter.^476^ Meanwhile, the U.S. Patent and Trademark Office (USPTO) has addressed a patent to the Tonix Pharmaceuticals company for its vaccine candidate namely TNX-801, which was designed to protect against smallpox and monkeypox.^477^ TNX-801 is a novel, horsepox-based live virus vaccine based on synthesized horsepox (sHPXV). Effectiveness and safety have been demonstrated both in mouse models previously.^478^ In addition, TNX-801 vaccination protects macaques from monkeypox challenge, no lesions were seen in all monkeys.^479^ The monkeypox virion membrane surface-binding protein E8L is vital for virus attachment to host cells, which may be also used to explore new vaccine against monkeypox.^480^ Importantly, knowledge of the immunological response and mechanism of monkeypox virus infection would be critical, providing new ideas for vaccine development. Identification of the genes responsible for the host-range defect of virus may engineer more effective and safe vaccines, such as C16L/B22R, C16L (MVA genome).^481^ For severely immunocompromised individuals, valid vaccination strategies that bypass CD4 negative cell help are needed to protect against virus.^482^

Global public health has been severely tested by the COVID-19 pandemic in the past two years. Confirmed cases of monkeypox are increasing rapidly worldwide especially in the US, which may have a negative impact on the global economy. With monkeypox being announced a public health emergency of international concern by WHO,^11^ people should raise awareness and build diagnostic capacity for the monkeypox epidemic in order to limit further spread of the virus. We should improve understanding and clinical management of monkeypox, as well as infection prevention and control skills, especially among public health personnel. At the same time, stigma and discrimination within the MSM community should be properly addressed and equitable access to treatment and vaccines should be ensured. Finally, we should initiate a global collaboration to conduct clinical investigations to test the efficacy and safety of monkeypox vaccines as well as antiviral drugs.
